# A Retrospective Study: Is Low-Intensity Pulsed Ultrasound (LIPUS) an Effective Alternate Treatment Option for Non-union?

**DOI:** 10.7759/cureus.29230

**Published:** 2022-09-16

**Authors:** Jacques Pretorius, Yousif Mohamed, Ahmed Mustafa, Nouman Nemat, Prasad Ellanti, Yasir Hammad, Tony Shaju, Sayed Nadeem

**Affiliations:** 1 Trauma and Orthopaedics, Letterkenny University Hospital, Letterkenny, IRL; 2 Trauma and Orthopaedic Surgery, St. James's Hospital, Dublin, IRL

**Keywords:** low-intensity pulsed ultrasound, delayed union, non-union, lipus, exogen

## Abstract

Background

There is ongoing controversy regarding the use of low-intensity pulsed ultrasound (LIPUS) therapy in patients with delayed union or non-union. Exogen (Bioventus, Durham, NC) is a well-known brand of LIPUS, and according to their data, 86% of non-union fractures will heal without the need for surgery. A few independent retrospective studies reported much lower healing rates.

Method

A retrospective observational study was performed assessing all the patients who underwent Exogen therapy in a single centre. All patients who were initiated on Exogen after three months with radiographic signs of the delayed union were included in the study. Routine follow-up appointments were organised until clinical and radiological healing could be confirmed. Daily 20-minute Exogen sessions were continued until the fracture was healed or up to a maximum of four months as recommended by the manufacturer.

Results

A total of 37 patients received Exogen therapy from 2012 to 2021, of which only 28 patients met our inclusion criteria and were subsequently analysed. The mean age of the patients was 52.0 (SD ± 20.2) with a male to female ratio of 1.7:1. The average time to healing was 115 (±51.2) days with a success rate of 82.14%. The average interfragmentary gap was 7.5 mm (±5.8) for the fractures that healed whereas the failed treatment was 16.1 mm (±13.8). There was no obvious association between outcomes after Exogen therapy and the patient’s age, sex, time to initiate Exogen, diabetes, and smoking status.

Conclusion

This study demonstrated a high success rate of LIPUS therapy for patients with delayed union and non-union. LIPUS represents a safe, non-invasive alternative to revision surgery. An independent risk factor for a potentially poor outcome is an increased interfragmentary gap.

## Introduction

Fracture non-union can lead to significant patient morbidity with poor quality of life. This remains a significant complication for fractures managed surgically or even conservatively [1,2]. Risk factors for delayed union/non-union can be classified as either patient dependent or independent. The patient-dependent risk factors include advanced age, gender, smoking, non-steroidal anti-inflammatory drugs (NSAIDs) use, metabolic disease, and nutritional deficiencies. Whereas patient independent factors include injury factors (fracture pattern, location, alignment, associated soft tissue injury, and degree of bone loss) and surgical factors (surgical technique and presence of infection) [3-5]. The definitive treatment for patients with non-union would be surgical management with osteosynthesis and autologous bone grafting; however, this can be associated with significant morbidity, a wide range of complications, and increased cost [6]. Therefore, there has been an increase in popularity in the use of alternative osteogenesis stimulation devices, which accelerates and promotes bone consolidation. These devices include induced currents, pulsed electromagnetic fields, and low-intensity pulsed ultrasound (LIPUS) [5].

LIPUS produces low-frequency pulsed ultrasound waves that act on the osteoprogenitor cells, which then produce more Cbfal/Rnx2 and osteocalcin, which is essential for osteogenesis [7]. Simultaneously it causes an increase in prostaglandin E2 and nitric oxide production, which encourages the tracking of inflammatory cells to the site of injury. This will subsequently stimulate osteoblast differentiation and proliferation, which will lead to new bone formation and increased bone healing [8].

The potential of these devices has been recognised by the National Institute for Health and Care Excellence (NICE), which states that it may be beneficial as an alternative treatment option for delayed union or non-union and has a potential for accelerating bone healing, while also admitting that there is still a lack of high-quality evidence available [9]. Exogen (Bioventus, Durham, NC) is a well-known brand of LIPUS, and according to their data, they have an 86% healing rate for fracture non-union [10]. A few independent retrospective studies reported much lower healing rates with rates as low as 47% [11,12]. With this uncertainty regarding the efficacy of LIPUS therapy still prevalent and the need for further evidence as highlighted by NICE, we assessed the efficacy of Exogen use for delayed union and non-union in our centre.

## Materials and methods

**Figure 1:**
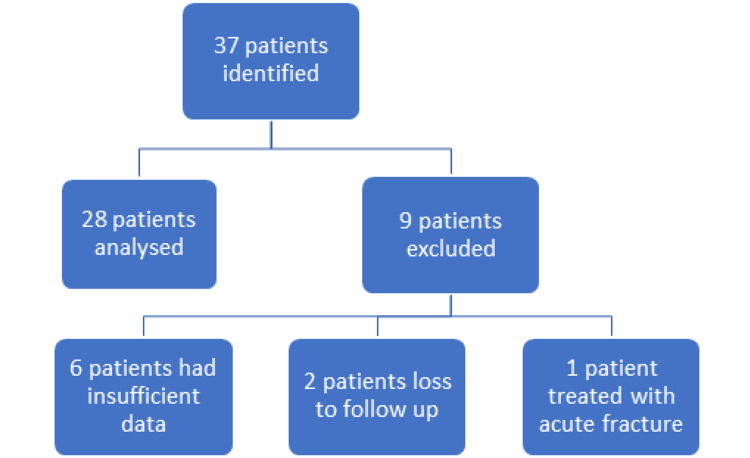
Patient selection with inclusion and exclusion criteria

This was a retrospective observational study performed in a single institution looking at all the patients who received Bioventus Exogen therapy from February 2012 to December 2021. The time to union of fractures is influenced by a variety of factors mentioned above but generally "delayed union" is said to occur when there is no radiological evidence of healing within three months of initial injury or surgery [9]. Whereas "non-union" is established when nine months have passed since the initial injury or surgery, with no visible signs of healing over the last three months [9]. Radiological evidence of non-union is defined as a lack of bridging callus in two or more cortices on two plain film orthogonal radiographs. In this study, we included all patients who received Exogen therapy for delayed union or non-union. Patients were excluded if they received Exogen before three months has passed since the initial injury or surgery, if they had pathological fractures, patients with infected cases, and patients with insufficient notes (Figure 1).

The treating orthopaedic consultant would decide to start Exogen therapy when they believe the patient will not heal due to specific risk factors or already has evidence of radiographic delayed union/non-union. The device would then be fitted in the clinic over the fracture site that is marked by the surgeon. Patients would then be directed by the Exogen representative to self-administer LIPUS daily for 20 minutes, at the same time frame until fracture union or up to four months, as advised by the manufacturer. The patients were followed up routinely until clinical and radiological union could be confirmed.

Radiological assessment was performed by two experienced orthopaedic surgeons during which the fracture gap was measured, as well as the radiological union, defined as three or more cortices bridged with a callus on two orthogonal views. The clinical union was defined as when the patient reported no functional pain, which was identified in the patient's clinical notes, along with the patient's demographics, comorbidities, date of Exogen application, and whether revision surgery was performed.

## Results

A total of 37 patients received Exogen therapy from 2012 to 2021, of which only 28 patients met our inclusion criteria. There were six patients with insufficient notes, two were lost to follow-up, and one patient started Exogen therapy for an acute fracture (Figure 1). The mean age of the patients was 52.0 (SD ± 20.2) years with a male to female ratio of 1.7:1. More than 50% of the patients had at least one comorbidity that could be a potential risk factor for developing non-union (Table 1).

**Table 1:** Socio-demographics of patients involved in the study LIPUS = low-intensity pulsed ultrasound.

Demographics		Union after LIPUS (n = 23)	Revision needed (n = 5)
Age	<50	13	2
	>50	10	3
Gender	Male	15	3
	Female	8	2
Diabetes	Yes	1	1
	No	22	4
Osteoporosis	Yes	5	2
	No	18	3
Bisphosphonates/steroids	Yes	1	1
	No	22	4
Smoker	Yes	3	2
	No	16	3
	Unknown	4	0

The average time to healing was 115 (±51.2) days after Exogen initiation with a success rate of 82.14% (23 out of 28 patients). This was confirmed on radiological assessment whereas the documentation regarding clinically healed fractures was poorly documented. The average interfragmentary gap was 7.5 mm (±5.8) for the fractures that healed, whereas the failed treatment was 16.1 mm (±13.8 mm). Most of the patients included in this study (24 of 28) had an atrophic non-union. The most common fracture sites leading to non-union were humerus shaft (n = 9) and tibia/fibula (n = 7) fractures (Table 2).

**Table 2:** Non-union characteristics

Site	Union	Non-union
Humerus	7	2
Forearm	4	0
Hand	1	0
Femur	4	2
Tibia/fibula	6	1
Foot	1	0
Type of non-union		
Atrophic	20	4
Hypertrophic	3	1
Interfragmentary gap	7.5 mm (SD ± 5.8)	16.1 mm (SD ± 13.8)

The patients that did not show any signs of healing received revision surgery at an average of 81.51 (±44.64) days. An average of 9.5 fracture clinic visits was recorded. Only seven patients received a computed tomography (CT) scan to confirm non-union and classify the sub-type. Initial surgical management was only performed in 50% of fractures, whereas the other 14 patients were treated conservatively with either casting or bracing. Patients that were treated conservatively tended to have a larger interfragmentary gap (Table 3).

**Table 3:** Fracture and treatment details of each participant DHS = dynamic hip screw; N/A = not applicable.

	Sex	Age	Bone segment	Primary treatment	Gap (mm)	CT	Type of non-union	Time to union from fitting (days)	Revision surgery
1	F	77	Femur	Nail	12	No	Atrophic	194	No
2	F	78	Humerus	Bracing	22	No	Atrophic	88	No
3	F	73	Tibia	Nail	1	No	Hypertrophic	145	No
4	M	48	Tibia/fibula	Plate	5	Yes	Atrophic	105	No
5	M	49	Humerus	Bracing	11	No	Atrophic	N/A	Yes
6	M	61	Tibia/fibula	Nail	7	No	Atrophic	N/A	Yes
7	M	36	Humerus	Bracing	19	No	Atrophic	56	No
8	M	48	Femur	Nail	5	No	Atrophic	91	No
9	F	85	Femur	Nail	4	No	Hypertrophic	N/A	Yes
10	M	48	Femur	Nail	9	No	Atrophic	203	No
11	F	17	Humerus	Bracing	7	Yes	Atrophic	152	No
12	M	69	Femur	DHS	0	Yes	Atrophic	125	No
13	F	71	Foot	Boot	9	No	Atrophic	35	No
14	F	31	Humerus	Nail	11	No	Atrophic	99	No
15	M	78	Tibia	Cast	9	No	Atrophic	156	No
16	M	53	Tibia/fibula	Nail	5	No	Atrophic	103	No
17	M	19	Ulna	Plate	4	Yes	Atrophic	182	No
18	F	76	Humerus	Bracing	1	No	Atrophic	104	No
19	M	40	Tibia/fibula	Plate	7	Yes	Atrophic	171	No
20	F	65	Scaphoid	Cast	3	Yes	Atrophic	84	No
21	M	47	Ulna	Cast	5	No	Hypertrophic	55	No
22	F	70	Humerus	Bracing	21	No	Atrophic	N/A	Yes
23	M	44	Humerus	Bracing	12	No	Atrophic	70	No
24	M	39	Humerus	Bracing	16	Yes	Atrophic	71	No
25	M	32	Femur	Nail	38	No	Atrophic	N/A	Yes
26	M	36	Radius/ulna	Plate	2	No	Atrophic	43	No
27	M	12	Radius/ulna	Cast	5	No	Hypertrophic	196	No
28	M	53	Tibia	Nail	3	No	Atrophic	122	No

## Discussion

**Figure 2:**
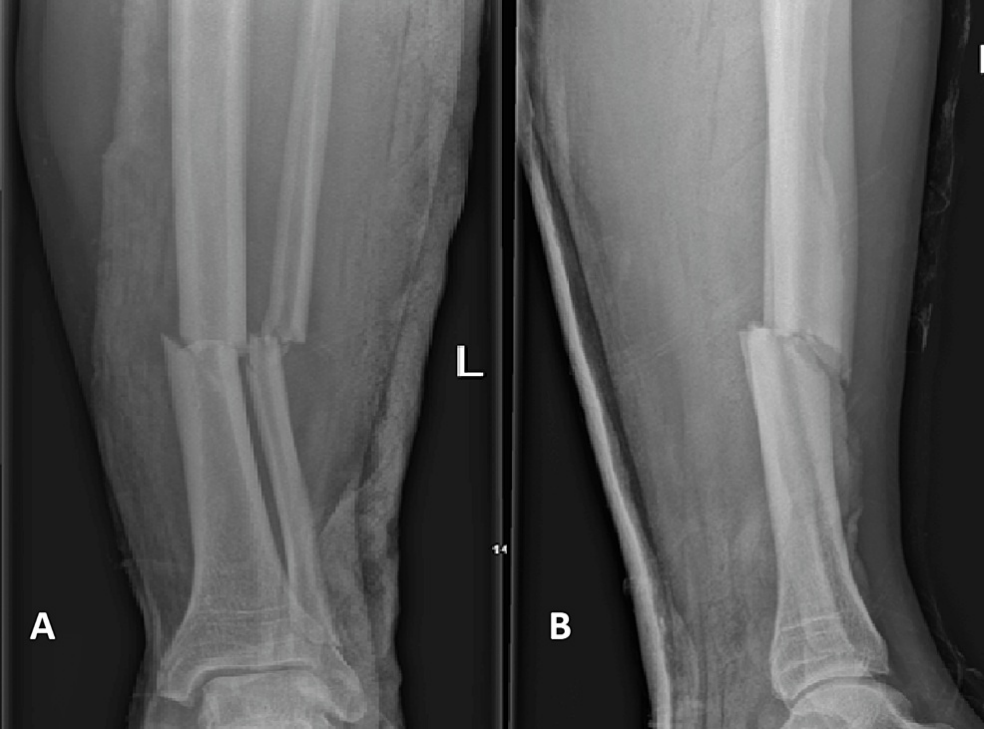
Anteroposterior (A) and lateral (B) radiographs of one of the cases in this study with a tibia/fibula fracture on presentation

**Figure 3:**
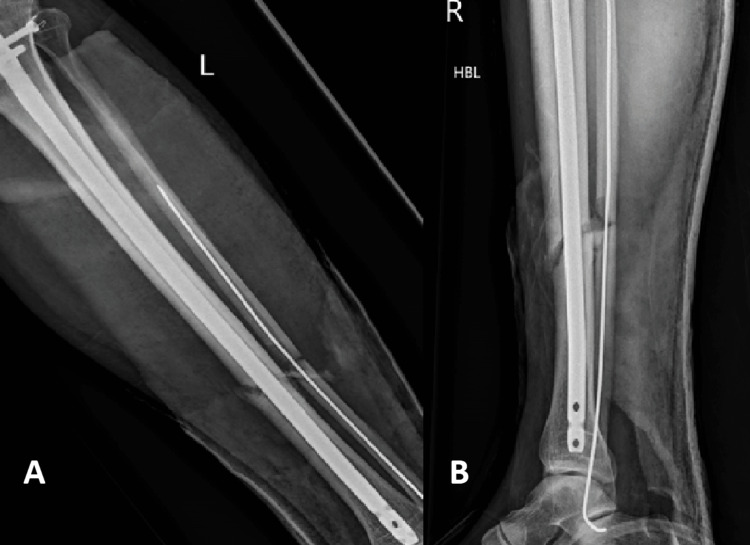
Anteroposterior (A) and lateral (B) radiographs of a tibia/fibula fracture after intramedullary nailing

**Figure 4:**
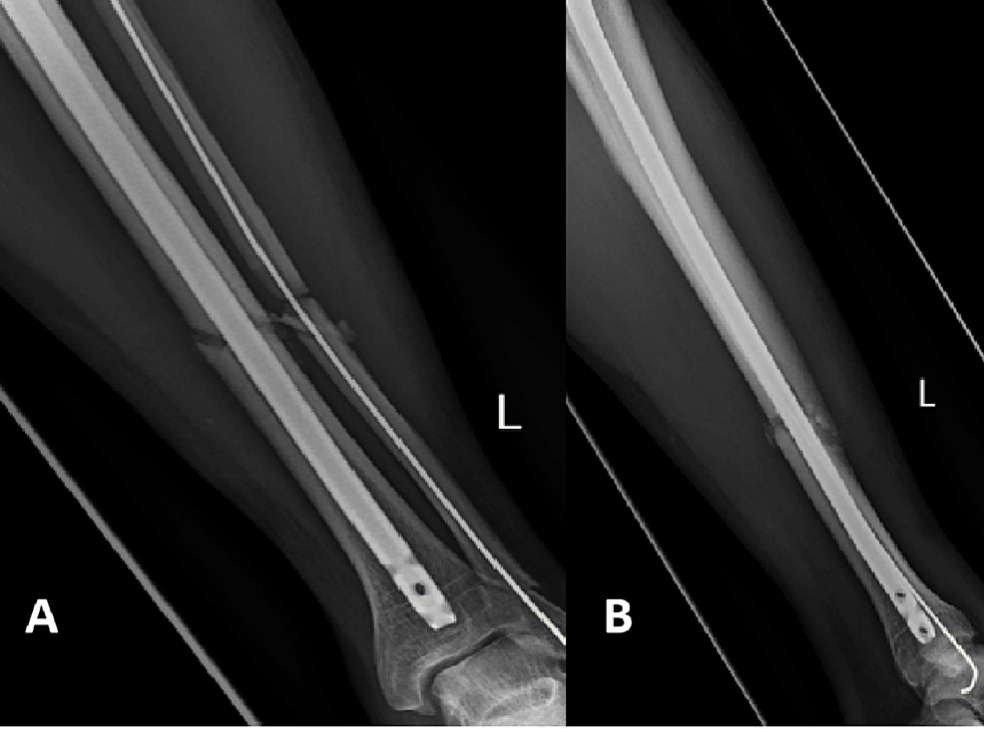
Anteroposterior (A) and lateral (B) radiographs of a tibia/fibula fracture after three months with radiological features of delayed union

**Figure 5:**
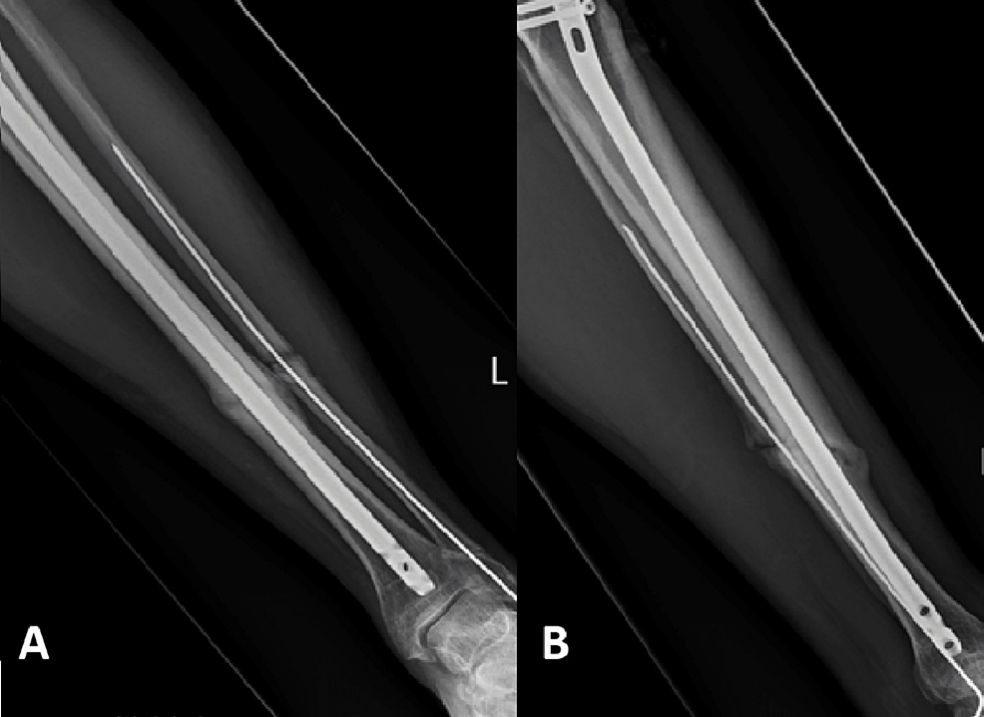
Anteroposterior (A) and lateral (B) radiographs of a tibia/fibula fracture after three months of Exogen therapy with radiological signs of union

The healing rate of patients using Exogen for delayed union in our study was 82.14%. This included patients who were treated with initial surgical fixation (79% union) as well as patients who were managed conservatively (86% union). This is in keeping with the healing rates according to the data provided by Exogen as well as other recently published studies, whose success rates vary between 78% and 90% [10,13-15]. An example of one of the successfully managed patients with LIPUS therapy can be appreciated in Figures 2-5. It is important to recognise that two recently performed retrospective reviews reported worse union rates with the lowest reporting a success rate of 47% [4,11,12].

The importance of interfragmentary bone gap as an independent risk factor for failing LIPUS therapy as demonstrated by Watanabe et al. was reiterated in this study [16]. The average gap was 16.1 mm in the patients who failed therapy in contrast to the 7.5 mm in the fractures that united successfully. It is even recommended by NICE guidelines, as well as Exogen, that LIPUS should ideally be used on fractures with an interfragmentary gap of less than 10 mm [9].

This study did not demonstrate any obvious association between smoking, diabetes, or age with failed LIPUS treatment, which is statistically supported by other available studies [2,12,17-19]. It has been clearly demonstrated that smoking has a definite negative impact on bone healing [2], but this does not seem to be the case when LIPUS therapy is used, as demonstrated in a meta-analysis performed by Leighton et al., who reported no association between smoking and failed LIPUS therapy [17]. A large retrospective, observational study that reviewed more than 4000 patients suggested that there is no association between healing rates in LIPUS therapy and patients' age [18]. Two recent retrospective studies performed did not demonstrate any association between the poor success rate of LIPUS therapy and the diagnosis of diabetes mellitus [12,19].

One of the major advantages of Exogen is the fact that it can be a cost-effective alternative to surgical management with the added advantage of being a non-invasive and low-risk intervention. NICE performed a literature review of Exogen therapy and established that successful treatment with Exogen instead of revision surgery can surmount cost savings of approximately £2407 per patient [9].

There are several limitations to this study, which include this being a retrospective, non-comparative study, with a small number of patients included, similar to other recently published studies (Table 4). The decision to initiate Exogen therapy, as well as subsequent follow-up visits, was not standardised, leading to a major disparity in the time to initiate Exogen therapy. The study also included multiple different fracture sites, fracture patterns, and initial treatment methods, which increases the number of variables that can influence the outcome significantly. The compliance rate of our patients was not documented, although the Exogen device routinely monitors compliance, which according to Exogen’s own available data demonstrates a high level of treatment compliance [10].

**Table 4:** Summary of key studies referenced in this article

Study	Fracture location	Treatment period (days)	Age (mean)	Patients Included	Time to Exogen (days)	Overall success rate
Teoh et al. (2018) [13]	5th metatarsal	75	39	30	101	90%
Bhan et al. (2021) [14]	Various bones	Up to 1 year	55	42	276	79%
Majeed et al. (2020) [15]	Various bones	178	57	47	492	79%
Hughes et al. (2022) [11]	Various bones	>120	N/A	58	263	47%
Adukia et al. (2021) [12]	Various bones	178	47	46	222	57%

## Conclusions

This study demonstrated a high success rate of LIPUS therapy for patients with delayed and non-union. This included a wide range of fracture sites, fracture patterns, and initial treatment options, which did not adversely affect the result. There was no association identified between union rates and diabetes, smoking, or age. An essential consideration to take into account prior to starting LIPUS therapy is the interfragmentary gap, as an increase in size is associated with poorer success rates.

Although there is still controversy regarding the effectiveness of LIPUS, it is undeniable at this point that it represents a low-risk, non-invasive alternative to revision surgery with multiple studies demonstrating promising results. Further research is nevertheless indicated in the form of high-quality large randomised controlled trials to more clearly demonstrate the role of LIPUS in delayed as well as non-union.
